# Prognostic factors of restrictive myopathy in thyroid eye disease

**DOI:** 10.1038/s41598-021-93275-9

**Published:** 2021-07-02

**Authors:** Jae Hwan Choi, Hoon Noh, Yoon-Duck Kim, Kyung In Woo

**Affiliations:** 1 Department of Ophthalmology, Samsung Medical Center, Sungkyunkwan University School of Medicine, 81 Irwon-ro, Gangnam-gu, Seoul, 06351 Korea; 2 Nune Eye Hospital, Seoul, Korea

**Keywords:** Health care, Prognosis

## Abstract

To investigate the prognostic factors of extraocular muscle restriction in patients with thyroid eye disease (TED), 65 patients with TED and restrictive myopathy were evaluated. Demographics, clinical activity score (CAS), smoking status, thyroid disease status, thyroid hormone status, thyroid autoantibody status, orbital computed tomography (CT) scan at initial presentation, and treatment regimens were assessed. The movements of the most severely affected extraocular muscles were categorized into five grades. The patients were divided into the improved and the not-improved group based on the improvement in the limitation of the extraocular muscle excursion (LOM) throughout the follow-up, and the groups were compared using clinical factors. The mean LOM significantly improved from 2.3 ± 1.1 to 1.7 ± 1.2 after 1 year of follow-up. The excursion of the most restricted muscle improved in 32 patients but not in 33 patients during the follow-up. The initial concentration of the thyroid-stimulating antibody (TSAb) was significantly lower in the improved (229.3 ± 114.1) than in the not-improved group (345.0 ± 178.6) (*P* = 0.02) Age, sex, smoking status, CAS, thyroid status, and muscle thickness on the CT scan did not significantly differ in the groups. This study showed that the initial concentration of TSAb is a factor affecting the recovery of restrictive myopathy.

## Introduction

Thyroid eye disease (TED) is a condition characterized by the inflammation of all periocular tissues, and it may result in proptosis, eyelid retraction, strabismus, or compressive optic neuropathy. The degree of clinical manifestations varies from asymptomatic or mild inflammation of the periocular tissue to severe visual dysfunction and eyelid to severe visual dysfunction. In addition, the clinical course of TED is occasionally chronic and relapsing; therefore, long-term care and follow-up are required.

During the active phase of TED in general, orbital fibroblasts, which have thyroid stimulating hormone receptor (TSHR) located in the orbital fat and rectus muscles, are activated by autoactivated T-cells and autoantibodies^1^. When activated, they interact with autoreactive T-cells and differentiate into myofibroblasts or mature adipocytes, depending on their cell subtypes and types of cytokines^2^. Fibroblasts also increase the production of hyaluronan leading to edematous swelling of soft tissues of the orbit^1,3^. Proinflammatory T-helper type 1 cytokines, such as interleukin 2 (IL-2), interferon gamma (IFN-γ), and tissue necrosis factor alpha are predominant during this phase; an inflammatory reaction is also dominant during this phase^4,5^. Therefore, immunosuppressive measures, including the administration of systemic steroids, immunosuppressants, and low-dose radiotherapy, have been used to suppress and shorten the active phase^6,7^. During the chronic fibrotic phase, T-helper type 2 (Th2) cytokines, such as IL-4, IL-5, and IL-13 are predominant^8^. They induce fibrotic changes in orbital soft tissues and rectus muscles by promoting collagen synthesis of fibroblasts^4,9^. Rehabilitation surgeries can be considered to correct structural changes resulting from the disease process.

Restrictive myopathy of the extraocular muscle occurs in approximately 20% of patients with TED^10^. It can be caused by rectus muscle swelling during an active phase and by fibrotic changes of the rectus muscles during a chronic phase. The most distressing symptom caused by restrictive myopathy is diplopia. It not only worsens the quality of life, but also causes work disability^11^. According to Nunery’s classification of extraocular movements, patients with TED having normal ocular motility and predominant lipogenic change were classified as type I, whereas patients with significant restrictive myopathy and diplopia within 20° of the primary position were classified as type II^12^. The type II disease, which also be called TED with significant restrictive myopathy, showed a higher peak age of onset, lower female-to-male ratio, and higher smoking prevalence than the type I disease. The clinical outcome was worse in patients with type II disease with a high incidence of compressive optic neuropathy and a poorer outcome after orbital decompression^12,13^.

As thyroid autoantibodies are known to play a role in pathogenesis and severity of TED^14–16^, other autoantibodies such as anti-calsequestrin 1 antibody (Anti-CASQ1 Ab) and anti-collagen XIII antibody (anti-COLXIII Ab) are also reported to be associated with the pathogenesis of TED^17,18^. Although the exact roles of these autoantibodies are still under investigation, calsequestrin 1 is found in extraocular muscle fibers and collagen XIII is in orbital fibroblasts^19,20^. Therefore, these antibodies might be related with the prognosis of restrictive myopathy.

Although the epidemiology and natural history of type II disease have been well elucidated, the clinical course of restrictive myopathy in patients with TED undergoing immunosuppressive treatment has not been well-described. Clinical factors affecting the restoration of the muscle and the strabismus angle during long-term follow-up have not been established.

In this study, we analyzed the clinical and laboratory features to identify the prognostic factors of TED-associated restrictive myopathy.

## Method

The medical records of patients who were diagnosed with TED and had diplopia within 20° from the primary position at the Samsung Medical Center between January 2011 and March 2016 were retrospectively reviewed. The study adhered to the tenets of the Declaration of Helsinki and was approved by the Institutional Review Board of Samsung Medical Center, Seoul, South Korea (IRB number 2017–02-090). Waiver of informed consent was permitted by the Institutional Review Board of Samsung Medical Center as the research was retrospective and anonymous presenting no threat to the rights and welfare of research subjects. All the enrolled patients were ethnically Korean. The patients with diplopia for more than 6 months before presentation or those who had undergone treatment for TED were excluded. The patients with compressive optic neuropathy or those who had been followed-up less than 6 months were also excluded.

The patient data, including demographics, thyroid status [triiodothyronine (T3), free thyroxine (fT4), and thyroid-stimulating hormone (TSH) levels], thyroid autoantibody status [thyrotropin receptor antibody (TSHR-Ab) and thyroid-stimulating antibody (TSAb) levels], and orbital computed tomography (CT) scans at the time of presentation, clinical activity score (CAS), limitation of extraocular muscle excursion (LOM), and diplopia grades and clinical features at the initial visit and 3, 6, 9, and 12 months of follow-up were assessed. The patients were divided into the improved or not-improved groups, based on the improvement of LOM of the most severely restricted rectus muscle at the last follow-up.

The TSHR-Ab titer was measured using a radioreceptor assay and the TRAK Human Kit (Brahms GmbH, Hennigsdorf, Germany). The TSAb concentrations were measured using the Thyretain (Diagnostic Hybrids Inc., Athens, OH, USA) bioreporter assay, according to the manufacturer's instructions.

The clinical findings of LOM, diplopia grade, and CAS were evaluated by a single oculoplastic specialist (KIW). The range of CAS was 0–7 points, based on the presence of seven signs of orbital inflammation.conjunctival injection, chemosis, eyelid redness, eyelid swelling, pain on eye movement, and retrobulbar pain^7^. The LOM was classified into five grades: grade 0, no restriction of muscle excursion; grade 1, mild restriction of muscle excursion (limitation of motion at extreme gaze); grade 2, moderate restriction (evident restriction, less than half of normal range); grade 3, severe restriction (evident restriction, more than half of normal range); grade 4, complete restriction (fixation of the globe). In addition, diplopia was also categorized using the Gorman grading: grade 0, no diplopia, grade 1, intermittent diplopia; grade 2, inconstant diplopia; grade 3, constant diplopia^21^.

Patients who presented with TED and normal thyroid function without any history of thyroid dysfunction were allocated to the euthyroid orbitopathy group in this study. The diagnostic criteria for this group included the elevation the concentration TSHR-Ab and/or TSAb, and clinical ocular features related to TED.

The maximum rectus muscle diameters were measured on the orbital CT scan by two investigators (JHC and HN). The maximum diameters of the superior and inferior rectus muscles were measured using a coronal scan, and those of the lateral and medial rectus muscles were measured using an axial scan^22^. The muscle was categorized as “enlarged” when the maximum diameter of the rectus muscle was higher than two standard deviations from the mean of the normal population in *Orgen & Ariyurek*’s study^23^.

The treatment modalities for the patients were not controlled and they underwent various treatments for TED: daily oral prednisolone (1 mg/kg/day) with tapering for 2–3 months, weekly intravenous (IV) methylprednisolone (500 mg/week for six times followed by 250 mg/week for six times), and/or orbital radiotherapy of 20 Gy. A group of patients underwent conservative treatment, and they were classified as the conservative group.

The Chi-squared test or Fisher’s exact test was used to analyze the categorical variables, such as age group, gender, smoking status, and treatment method. The student’s t test or Mann–Whitney test was used to compare the numerical variables of the improved and not-improved groups. To investigate the factors associated with the prognosis of restrictive myopathy associated with TED, univariate and multivariate logistic regression analyses were performed. The parameters with a P value of less than 0.2 in the univariate analysis, age, and sex ratio were included for the multivariate logistic analysis. To evaluate interobserver (measured by JHC and HN) reproducibility for the measurement of the maximum rectus muscle diameters, the intraclass correlation coefficients (ICCs) were calculated. All statistical analyses were performed using R software version 3.6.3 (R Foundation for Statistical Computing, Vienna, Austria). A *P* value of < 0.05 was considered statistically significant.

## Result

Of the 652 patients diagnosed with TED during the study period, 65 met the inclusion criteria for restrictive myopathy. Their mean age was 54.0 ± 10.0 (range 29–76) years. For these 65 patients, the female-to-male ratio was 0.85:1, whereas it was 3.88:1 for all 652 patients with TED. The mean duration of diplopia before presentation was 4.3 ± 3.8 months.

Regarding smoking status, 48 patients (73.8%) had never smoked, 3 (4.6%) were past smokers, and 14 (21.6%) were current smokers during the first visit. In 587 TED patients without restrictive myopathy, 494 (84.2%) had never smoked, 39 (6.6%) were past smokers, and 54 (2.4%) were current smokers, respectively. The smoking status of patient with restrictive myopathy was significantly different from that of patients without restrictive myopathy (*P* < 0.01).

Thyroid status at presentation with TED included euthyroid in 47 patients (72.3%), hypothyroid in 5 patients (7.7%), and hyperthyroid in 13 patients (20.0%).

The following treatments were administered: IV methylprednisolone in 52 (80.0%) cases, oral prednisolone in 23 (35.4%) cases, and orbital radiotherapy in 23 (35.4%) cases. Seven patients (10.8%) had to undergo strabismus surgery after the stabilization of the clinical activity and degree of deviation angle. The mean follow-up duration was 11.4 ± 2.4 (range 6–15) months (Table 1).

**Table 1:** Comparisons of demographic and clinical characteristics in the improved and not-improved groups at initial presentation.

Characteristics	All patients	Improved (N = 32)	Not-improved (N = 33)	*P* value
N (%)	65 (100%)	32 (49.2%)	33 (50.8%)	NA
Mean age, years (SD)	54.0 (10.0)	54.6 (8.1)	53.3 (8.1)	0.62^†^
**Age group, n (%)**				
≤ 50 years	18 (27.7%)	7 (21.9%)	11 (33.3%)	0.30^‡^
> 50 years	47 (72.3%)	25 (78.1%)	22 (66.7%)	
Sex, M:F	35 : 30	17 : 15	18 : 15	0.91^‡^
Mean symptom onset, mo (SD)	4.3 (3.8)	3.6 (2.0)	4.9 (4.8)	0.16^†^
**Smoking status, n (%)**				
Non smoker	48 (73.8%)	21 (65.6%)	27 (81.8%)	0.37^‡^
Past smoker	3 (4.6%)	2 (6.3%)	1 (3.0%)	
Current smoker	14 (21.6%)	9 (28.1%)	5 (15.2%)	
Mean LOM (SD)	2.3 (1.1)	2.5 (1.0)	2.2 (1.1)	0.25^†^
**Diplopia grade, N (%)**				
Grade 0	0 (0.0%)	0 (0.0%)	0 (0.0%)	0.59^‡^
Grade 1	27 (41.5%)	15 (46.9%)	12 (36.4%)	
Grade 2	13 (20.0%)	5 (15.6%)	8 (24.2%)	
Grade 3	25 (38.5%)	12 (37.5%)	13 (39.4%)	
Mean CAS (SD)	2.9 (1.7)	3.0 (1.9)	2.7 (1.6)	0.55^†^
**Mean thyroid hormone, autoAb**				
TSH, μIU/mL (SD)	2.6 (6.5)	2.7 (7.4)	2.5 (5.6)	0.89^†^
T3, ng/dL (SD)	125.7 (39.8)	121.2 (40.6)	129.5 (39.6)	0.49^†^
fT4, ng/dL (SD)	1.5 (1.2)	1.7 (1.6)	1.3 (0.6)	0.18^†^
TSAb, % (SD)	288.6 (159.9)	229.3 (114.1)	345.0 (178.6)	0.02^†^
TSHR-Ab, IU/L (SD)	24.5 (43.0)	24.3 (54.7)	24.7 (29.9)	0.98^†^
**Presence of thyroidopathy, n (%)**				
Euthyroid orbitopathy	10 (15.4%)	5 (15.6%)	5 (15.2%)	1.00^‡^
Orbitopathy with thyroidopathy	55 (84.6%)	27 (84.4%)	28 (84.8%)	
**Thyroid status at visit, n (%)**				
Euthyroid	47 (72.3%)	25 (78.1%)	22 (66.7%)	0.45^‡^
Hypothyroid	5 (7.7%)	1 (3.1%)	4(12.1%)	
Hyperthyroid	13 (20.0%)	6 (18.8%)	7(21.2%)	
Treatment, n (%)*				
Conservative	4 (6.2%)	2 (6.3%)	2 (6.1%)	1.00^‡^
Oral prednisolone	22 (33.8%)	11 (34.4%)	11 (33.3%)	0.27^‡^
IV methylPD	52 (80.0%)	27 (84.4%)	23 (69.7%)	0.16^‡^
Orbital radiotherapy	23 (35.4%)	11 (34.4%)	12 (36.4%)	0.87^‡^
Strabismus operation	7 (10.8%)	0 (0.0%)	7 (21.2%)	NA

*N* number, *SD* standard deviation, *Mo* month, *M* male, *F* female, *LOM* limitation of muscle excursion, *CAS* clinical activity score, *TSH* thyroid stimulating hormone, *T3* triiodothyronine, *fT4* free thyroxine, *TSAb* thyroid-stimulating antibody, *TSHR-Ab* thyroid stimulating hormone receptor antibody, *IV methylPD* intravenous methylprednisolone, *NA* not available,

^†^Student’s t test or Mann–Whitney test, ^‡^Chi-square test or Fisher’s exact test.

*If the patient underwent multiple treatment modalities, all modalities were included.

After 1 year of treatment and follow-up, the mean CAS value improved from 2.9 ± 1.7 to 1.2 ± 1.5 (*P* < 0.01). The mean LOM also improved from 2.3 ± 1.1 to 1.7 ± 1.2, which was statistically significant (*P* < 0.01). The mean diplopia grade decreased from 2.0 ± 0.9 to 1.6 ± 1.1, but it was not significant (*P* = 0.07). The mean fT4 and TSH concentrations did not change significantly (*P* = 0.55 and 1.00, respectively). The mean TSAb concentration decreased from 288.8 ± 157.9% to 256.2 ± 103.5%, but it was not statistically significant (*P* = 0.56).

Although the average value of LOM decreased throughout the follow-up period, there was a group of patients (33 patients, 50.8%) who showed no improvement in LOM. The clinical factors of the improved (32 patients, 49.2%) and not-improved (33 patients, 50.8%) groups were compared to identify the factors affecting the improvement in LOM. There were no differences in the demographics, smoking status, mean CAS, thyroid status and thyroid hormone levels at visit, time to symptom onset, and presence of thyroidopathy. The mean initial TSAb concentration was 229.3 ± 114.1% in the improved group and 345.0 ± 178.6 in the not-improved group; the not-improved group showed a 66.4% higher initial TSAb concentration than the improved group. The initial TSAb concentration was significantly higher in the not-improved group than in the improved group (*P* = 0.02). The initial TSHR-Ab concentration was not significantly different between two groups. The analysis of the treatment modalities showed that there was no significant difference between the treatment regimens used in the two groups. The proportion of patients receiving IV methylprednisolone treatment was slightly higher (84.4%) in the improved group than in the not-improved group (69.7%); however, the difference was not statistically significant (*P* = 0.16).

The extraocular rectus muscle diameter analysis using the orbital CT scan showed that the most frequently involved muscle was the inferior recuts muscle (42/130), followed by the medial rectus muscle (38/130). There was no significant difference between the initial mean maximum diameters of the rectus muscles in the improved and not-improved groups. The mean number of enlarged rectus muscles was 2.5 ± 1.6 in the improved group and 2.7 ± 1.9 in the not-improved group (*P* = 0.62). The mean total maximum diameter of the 8 rectus muscles was 35.2 ± 7.0 mm in the improved group and 35.0 ± 7.8 mm in the not-improved group (*P* = 0.93). The orbital CT analysis showed that the intraobserver and interobserver intraclass correlation coefficient (ICC) showed good agreement of the assessments of the maximum diameters of the rectus muscles. The intraobserver ICC ranged from 0.91 to 0.95, and the interobserver ICC ranged from 0.90 to 0.92.

In the univariate logistic regression analysis, a higher initial TSAb concentration was associated with poor prognosis in the improvement of LOM throughout the follow-up period (*P* = 0.03). The multivariate logistic regression analysis showed that higher TSAb levels were also associated with poor recovery of LOM throughout the follow-up period (*P* = 0.04). The fT4 concentration tended to be lower in the not-improved group, but the difference was not statistically significant (*P* = 0.08) (Table 2).

**Table 2:** Logistic regression analysis of the pre-treatment variables associated with poor prognosis of extraocular muscle movement limitation in the restrictive myopathy associated with thyroid eye disease.

Parameters	Univariate analysis			Multivariate analysis		
	OR	95% CI	*P*	OR	95% CI	P*
Age, years	0.99	0.94–1.04	0.614	0.91	0.81–1.03	0.138
Sex, M:F	0.94	0.36–2.51	0.909	0.55	0.30–9.94	0.546
Disease onset, mo	1.11	0.95–1.31	0.191	1.01	0.74–1.37	0.953
CAS	0.91	0.68–1.22	0.540			
Diplopia grade	1.12	0.66–1.90	0.681			
TSH, μIU/mL	0.99	0.91–1.08	0.89			
T3, ng/dL	1.01	0.99–1.02	0.483			
fT4, ng/dL	0.61	0.25–1.45	0.259	0.19	0.03–1.18	0.075
TSAb, 10%	1.06	1.01–1.11	**0.028**	1.09	1.00–1.18	**0.044**
TSHR-Ab, 10 IU/L	1.00	0.89–1.14	0.975			
**Treatment, n**						
Conservative	0.97	0.13–7.32	0.975			
Oral PD	0.96	0.34–2.67	0.929			
IV methylPD	0.43	0.13–1.43	0.166	0.80	0.11–5.81	0.827
Radiotherapy	1.09	0.39–3.02	0.867			

*mo* month, *M* male, *F* female, *CAS* clinical activity score, *TSH* thyroid stimulating hormone, *T3* triiodothyronine, *fT4* free thyroxine, *TSAb* thyroid-stimulating antibody, *TSHR-Ab* thyroid stimulating hormone receptor antibody, *n* number, *IV methylPD* intravenous methylprednisolone, *CI* confidence interval, *OR* odds ratio.

*Variables with *P* < 0.20 in the univariate analysis, age, and sex were included in the multivariate analysis.

For the longitudinal analysis, the mean LOM at initial presentation did not differ in the improved and the not-improved groups; however, the difference became obvious after 9 months of follow-up. The mean diplopia grade began to improve significantly in the improved group after 3 months of follow-up (Table 3). The mean TSAb at 12 months was lower in the improved group, but the difference was not significant (*P* = 0.13). The mean CAS, fT4, TSH, and TSHR-Ab concentrations were not significantly different in the two groups during follow-up.

**Table 3:** Comparisons of longitudinal changes in the limitation of extraocular movement, diplopia, clinical activity score, and thyroid status in the improved and not-improved groups.

	Pre-treatment	3 months	6 months	9 months	12 months
**LOM (SD)**					
Improved	2.5 (1.0)	2.2 (1.0)	1.9 (1.1)	1.2 (1.1)^†^	1.2 (1.0)^†^
Not-improved	2.2 (1.1)	2.4 (0.9)	2.2 (1.1)	2.4 (1.0)^†^	2.3 (1.1)^†^
**Diplopia grade (SD)**					
Improved	1.9 (0.9)	1.3 (0.8)^†^	1.3 (0.7)^†^	1.3 (1.0)^†^	1.1 (1.0)^†^
Not-improved	2.0 (0.9)	2.2 (0.9)^†^	2.1 (0.9)^†^	2.2 (0.8)^†^	2.1 (1.0)^†^
**CAS (SD)**					
Improved	3.0 (1.9)	1.3 (1.6)	1.5 (1.5)	1.2 (1.2)	1.4 (1.7)
Not-improved	2.7 (1.6)	1.6 (1.4)	1.7 (1.2)	1.7 (1.5)	1.1 (1.4)
**TSH, μIU/mL (SD)**					
Improved	2.7 (7.4)	2.7 (7.0)	1.8 (3.0)	0.6 (1.0)	3.3 (4.4)
Not-improved	2.5 (5.6)	5.7 (11.6)	4.3 (7.0)	0.6 (0.9)	3.6 (5.9)
**T3, ng/dL (SD)**					
Improved	121.2 (40.6)	146.1 (70.3)	106.6 (22.8)	111.6 (28.5)	97.1 (19.4)^†^
Not-improved	129.5 (39.6)	111.1 (27.7)	120.0 (32.6)	130.8 (38.2)	127.1 (30.2)^†^
**fT4, ng/dL (SD)**					
Improved	1.7 (1.6)	1.3 (0.3)	1.3 (0.3)	1.3 (0.3)	1.3 (0.6)
Not-improved	1.3 (0.6)	1.1 (0.2)	1.1 (0.2)	2.3 (1.9)	1.5 (0.7)
**TSAb, % (SD)**					
Improved	229.3 (114.1)	NA	NA	NA	162.5 (116.6)
Not-improved	345.0 (178.6)	NA	NA	NA	266.4 (118.2)
**TSHR-Ab, IU/L (SD)**					
Improved	24.3 (54.7)	14.6 (22.9)	6.5 (12.5)	9.6 (12.8)	3.2 (3.1)
Not-improved	24.7 (29.9)	15.3 (26.9)	4.7 (5.3)	2.1 (2.0)	7.0 (11.6)

*LOM* limitation of muscle excursion, *CAS* clinical activity score, *TSH* thyroid stimulating hormone, *T3* triiodothyronine, *fT4* free thyroxine, *TSAb* thyroid-stimulating antibody, *TSHR-Ab* thyroid stimulating hormone receptor antibody.

^†^The difference between the two groups was significant (*P* < 0.05, Student’s t test or Mann Whitney U test).

## Discussion

In this study, the mean LOM in patients with TED significantly improved throughout the follow-up after treatment; however, a group of patients showed no improvement in LOM. A higher initial titer of TSAb was associated with a poor prognosis for the recovery of LOM whereas initial TSHR-Ab level was not related to the prognosis of LOM. The mean initial TSAb concentration of the not-improved group was 66.4% higher than that of the improved group. In addition, the diplopia grade at 1 year after treatment was significantly lower in the improved group than in the not-improved group, while the pre-treatment diplopia grade, pre-treatment LOM, and mean rectus muscle thickness did not significantly differ in the two groups.

The mean age at the initial visit was 54.6 years in male patients and 53.3 years in female patients in this study. It was much higher than the mean age (42.8 years in men and 41.7 years in women) of Korean patients with dysthyroid TED from the multicentral epidemiological study involving patients in 24 centers^24^. This was consistent with other studies that reported a significantly higher mean age in patients with restrictive myopathy^12,25^. The female-to-male ratio was 0.85:1 for restrictive myopathy in this study. This contrasted with the female-to-male ratio of all 652 patients with TED during the study period (3.88:1). In Woo et al.’s Korean multicenter study, the female-to-male ratio (3.9:1) was compatible with that of all the 652 patients. The female-to-male ratio of patients with restrictive myopathy has been reported to be lower (1–1.74:1) than that of all patients with TED^26–28^.

According to several studies, smoking is strongly associated with the development of TED and unfavorable clinical outcomes. Current smokers had more than twice the odds ratio or relative risk of TED development than those who had never smoked or past smokers. Current smokers also showed a higher incidence of proptosis, diplopia, and total ophthalmopathy, but no differences in clinical manifestations and clinical outcomes in some reports^29^. In our study, the ratio of current smoker was higher in TED patients with restrictive myopathy, compared to patients without restrictive myopathy. However, the smoking factor was not related to the prognosis of restrictive myopathy, which contrasted with other studies reporting worse ocular motility prognosis in smokers^30,31^. The limitations of our study were the small sample size and the inclusion of only the initial smoking status.

Of note, the proportion of patients with euthyroid orbitopathy was 15.4%. This was higher than that of other epidemiologic studies conducted more than a decade ago: 5.0–7.3% of euthyroid orbitopathy patients with TED of all the stages^32–34^. According to a study by Yoon et al. the proportion of euthyroid orbitopathy was 14.7%, and euthyroid orbitopathy was known to take a milder form of TED, although the prevalence of restrictive myopathy was not significantly different from that of hyperthyroid TED^35^. Euthyroid orbitopathy was diagnosed when typical clinical signs of TED and high autoantibody titers of TSHR-Ab or TSAb were observed without any evidence of preceded dysthyroid disease. The higher proportion of euthyroid orbitopathy in our study may be related to the recent trend of routine autoantibody testing for the diagnosis of TED. Further studies are needed to establish the relationship between euthyroid orbitopathy and restrictive myopathy.

Muscle thickness is known to be related to the degree of extraocular muscle motility restriction in patients with active-phase TED^22^. The correlation between the cross-sectional area of the vertical rectus muscles in the CT scan and the vertical angle of deviation of the eye was previously reported^36^. However, in search of prognostic factors of restrictive myopathy on the pre-treatment CT scans, we could not find any significant factors. The type and number of involved muscles and the muscle thickness at initial presentation did not affect the recovery of the limitation of muscle movement. Since initial pre-treatment CT scans were only used in this study, imaging studies compatible with the 1-year clinical status could not be evaluated in this study.

Since all the patients in this study met the moderate-to-severe TED classification by EUGOGO, the treatment recommendations of EUGOGO were adopted^7^. Eighty percent of patients received IV methylprednisolone as a first-line treatment after a thorough discussion of the treatment course and side effects. Patients who could not follow the treatment schedule took oral steroid treatment as the first treatment. Orbital radiotherapy and oral prednisolone treatments were administered to 35.4% and 33.8% of the patients, respectively (Table 1). The proportion of patients who received IV methylprednisolone was higher in the improved group, but the difference was not significant (*P* = 0.16). Although there are debates on the efficacy of orbital radiotherapy for TED, it has been accepted as an effective treatment option for restrictive myopathy related to TED^7^. However, in our study, the proportion of patients who underwent radiotherapy in the improved and not-improved groups were not different. A limitation of our study is that a few patients received radiation therapy, and there may have been a selection bias for treatment decisions.

During the longitudinal analysis, the treatment outcomes were evaluated chronologically (Table 3). Notably, the LOM of the improved group did not show any improvement until after 9 months of follow-up, whereas the CAS in both groups decreased after 3 months of follow-up. This implies that the restoration of LOM takes time after treatment even though the inflammatory signs decrease during the early post-treatment period. Therefore, we need to follow-up on patients with restrictive myopathy for a long time after immunosuppressive treatment to determine the effect of treatment. Interestingly, the diplopia grade decreased in the improved group after 3 months of follow-up: quality of vision can improve from the early post-treatment period even if LOM does not recover at all. Gorman diplopia scale is based on the severity of diplopia in the primary position^21^, so it is closely related to severity of strabismus and fusion ability at the primary position. However, LOM scale in this study was based on the maximal gaze potential of each rectus muscle, which did not reflect severity of strabismus at the primary position. We considered this was the reason of discordance between LOM and diplopia grade changes during follow-up.

Some studies have categorized the severe TED subtypes and demonstrated the association of TSHR-Ab and TSAb with the severity of restrictive myopathy in TED^25,37^. Our study is worthwhile because it focused solely on the prognosis of restrictive myopathy. A high titer of TSAb may be predictive of a poor prognosis for restrictive myopathy, as our study shows; moreover, if the titer of TSAb does not diminish through the follow-up, it may also contribute to a poor prognosis in restrictive myopathy^38^. Therefore, checking the concentration of TSAb during follow-up may be important for prognosticating restrictive myopathy.

TSHR-Ab, TSAb and other autoantibodies activate helper T-cells, which, in turn, activate B-cells to express autoantibodies or become autoactivated T-cells^14,16^. It has been generally acknowledged that thyroid autoantibodies plays a critical role on the pathogenesis and clinical course of TED^1,15,16,39^. However, there are several reports disputing the relationship between thyroid autoantibodies and TED, since there were a group of TED patients with negative TSHR-Ab or TSAb^40–42^. In addition, TSH receptor is not exclusively expressed in the orbital soft tissue and thyroid, and it can be also expressed in the soft tissue in other sites, such as abdominal wall not affected by TED^43^. These reports called conventional knowledges in the role of TSHR-Ab on TED into questions.

Several researches demonstrated that anti-CASQ1 Ab and anti-COLXIII Ab were specific to TED^17,18^. CASQ1 is expressed in skeletal muscles including extraocular muscles and COLXIII is expressed in orbital fibrocytes. The expression of CASQ1 in the extraocular muscles is 4.8 times higher than that in other skeletal muscles, and anti-CASQ1 Ab is detected in 92% of TED patients showed extraocular muscle involvement^17,18,44^. Since CASQ1 is related to regulation of cytoplasmic calcium ion level in skeletal muscles, anti-CASQ1 Ab may disrupt normal skeletal muscle function by disturbing calcium ion regulation of skeletal muscles^19^. In these reasons anti-COLXIII Ab and anti-CASQ1 Ab are plausible candidates for prognostic factors of restrictive myopathy of TED. For now, however, there is no research investigated the prognostic value of these autoantibodies or their relationship with TSAb or TSHR-Ab in the pathogenesis of the TED. Additional study on these antibodies is warranted.

This study was designed as a retrospective study and performed at a single tertiary care center in Korea. The limitations of this study are referral bias, uncontrolled treatment options, and the small sample. Further multicenter studies with larger sample sizes may be required to confirm our findings.

In conclusion, patients with TED and restrictive myopathy who had higher pre-treatment TSAb titers showed poorer responses to treatment. A further study on other autoantibodies such as anti-COLXIII and anti-CASQ1 Abs is warranted to evaluate their association on prognosis of restrictive myopathy. Initial thyroid function, demographic features such as age, sex, symptom onset, and initial rectus muscle thickness did not relate to the clinical course of restrictive myopathy.
